# Geographic population structure of the African malaria vector *Anopheles gambiae* suggests a role for the forest-savannah biome transition as a barrier to gene flow

**DOI:** 10.1111/eva.12075

**Published:** 2013-06-10

**Authors:** J Pinto, A Egyir-Yawson, JL Vicente, B Gomes, F Santolamazza, M Moreno, JD Charlwood, F Simard, N Elissa, D Weetman, MJ Donnelly, A Caccone, A della Torre

**Affiliations:** 1 Unidade de Parasitologia Médica, Centro de Malária e outras Doenças Tropicais, Instituto de Higiene e Medicina Tropical, Universidade Nova de LisboaLisbon, Portugal; 2 Biotechnology and Nuclear Agriculture Research Institute, Ghana Atomic Energy CommissionLegon, Ghana; 3 Dipartimento di Sanità Pubblica e Malattie Infettive, Istituto Pasteur-Fondazione Cenci-Bolognetti, Università di Roma “La Sapienza”Rome, Italy; 4 Division of Infectious Diseases, School of Medicine, University of California San DiegoLa Jolla, CA, USA; 5 Vector Group, Liverpool School of Tropical MedicineLiverpool, UK; 6 MIVEGEC (Maladies Infectieuses et Vecteurs: Ecologie, Genetique, Evolution et Contrôle), UMR IRD224-CNRS5290-UM1-UM2, Institut de Recherche pour le DéveloppementMontpellier, France; 7 Unité d'Entomologie Médicale, Institut Pasteur de MadagascarAntananarivo, Madagascar; 8 Department of Ecology and Evolutionary Biology, Yale UniversityNew Haven, CT, USA

**Keywords:** *Anopheles gambiae*, geographic regions, microsatellites, molecular forms, population structure

## Abstract

The primary Afrotropical malaria mosquito vector *Anopheles gambiae sensu stric*to has a complex population structure. In west Africa, this species is split into two molecular forms and displays local and regional variation in chromosomal arrangements and behaviors. To investigate patterns of macrogeographic population substructure, 25 *An. gambiae* samples from 12 African countries were genotyped at 13 microsatellite loci. This analysis detected the presence of additional population structuring, with the M-form being subdivided into distinct west, central, and southern African genetic clusters. These clusters are coincident with the central African rainforest belt and northern and southern savannah biomes, which suggests restrictions to gene flow associated with the transition between these biomes. By contrast, geographically patterned population substructure appears much weaker within the S-form.

## Introduction

Many studies have attempted to identify genetic discontinuities between conspecific populations and to determine the factors that promote differentiation. This is a critical step for predicting the evolution of populations under different scenarios, including those that involve human-made environmental changes (Crispo et al. 2011). In medically important insects, the evolutionary relevance of these predictions gains a public health dimension, as they can be used to model the dispersal of genes of interest such as those related to insecticide resistance or refractoriness to infection by pathogens (Donnelly et al. 2002).

The nominal species of the *Anopheles gambiae* Giles complex (Diptera: Culicidae), *Anopheles gambiae sensu stricto* (hereafter termed ‘*An. gambiae’*) is a primary vector of human malaria in Africa. It is widely distributed throughout sub-Saharan Africa in close association with humans. There is evidence that this species is undergoing a process of incipient speciation. The speciation process appears to be restricted to west Africa and involves sympatric populations. Initially, heterogeneities have been found in the distribution of paracentric inversions at chromosome 2, which displayed strong heterokaryotype deficits. This led to the description of five chromosomal forms (cytoforms) each in Hardy–Weinberg equilibrium and characterized by distinct arrangements of inversions (Coluzzi et al. 1979, 2002). Following the early recognition of the five cytoforms, the species was tentatively split into two molecular forms, denoted M and S, identified by RFLP patterns in the X-linked ribosomal DNA (rDNA) intergenic spacer (IGS) (Favia et al. 1997; della Torre et al. 2001, 2002). The S-form has a continent-wide distribution, whereas the M-form appears to be confined to west Africa where it commonly occurs in sympatry with the S-form (della Torre et al. 2005). However, despite the extensive area of sympatry, MS hybrids are rarely seen (della Torre et al. 2005; Simard et al. 2009), with the exception of the extreme west of Africa (Caputo et al. 2008; Oliveira et al. 2008).

Initial genome-wide genotyping analyses revealed that differentiation between molecular forms was restricted to relatively small genomic regions located on the three chromosomes (Turner et al. 2005; White et al. 2010). More recently, however, whole-genome analyses based on next-generation sequencing and SNP microarrays have shown that M/S differentiation is more widespread across the genome than previously thought (Lawniczak et al. 2010; Neafsey et al. 2010; Weetman et al. 2010). Subsequently, the detection of genomic islands of divergence was found to be influenced by the degree of realized gene flow between the forms, which varies across west Africa (Weetman et al. 2012). As gene flow decreases, differentiation across the genome tends to increase and masks the initial divergent genomic regions. These findings point to a case of sympatric ecological speciation under divergent selection within *An. gambiae* (Diabaté et al. 2008; Costantini et al. 2009).

The phenotypic repercussions of the genetic divergence between molecular forms are still unresolved. Recent studies have shown that M-form larvae outcompete the S-form in the presence of predators, which may contribute to habitat segregation observed between forms (Diabaté et al. 2008; Gimonneau et al. 2010). M-form larvae prevail in areas with more permanent breeding sites (hence with higher predator pressure), whereas the S-form predominates in temporary rain-dependent breeding sites, perhaps due to a superior competitive ability where predation pressure is lower (Gimonneau et al. 2012). Genetic divergence between molecular forms may also impact both malaria transmission and vector control. A variant of the complement-like protein TEP1 with anti-parasitic activity was found to be fixed in M-form but absent in sympatric S-form populations of Mali and Burkina Faso (White et al. 2011). This was the first evidence of how subdivision within *An. gambiae* may affect vector competence. Another striking example comes from the contrasting differences in the frequency of knockdown resistance (*kdr*) mutations found between molecular forms. In spite of widespread sympatry between M- and S-forms, for a decade following their discovery in *An. gambiae* (Martinez-Torres et al. 1998), *kdr* mutations were found at high frequency in S-form populations but were rare in M-form (Santolamazza et al. 2008). Only recently, these mutations are becoming more common in M-form populations (Dabiré et al. 2009; Lynd et al. 2010).

In addition to the M- and S-forms partitioning, there is evidence for further population substructure within each of the molecular forms of *An. gambiae*. Microsatellite and AFLP analyses of S-form populations belonging to the SAVANNA and BAMAKO cytoforms revealed significant differentiation between these cytoforms in Mali (Taylor et al. 2001; Slotman et al. 2006). Similarly, Slotman et al. (2007) reported significant genetic differentiation between M-form populations of the FOREST and MOPTI cytoforms from Cameroon and Mali, respectively. These results led the authors to hypothesize that the M-form may actually consist of two partially isolated entities (Slotman et al. 2007; Lee et al. 2009).

With some exceptions (e.g. Lehmann et al. 2003; della Torre et al. 2005; Esnault et al. 2008; Choi and Townson 2012), the complex scenario of population subdivision within *An. gambiae* has been evidenced by studies that have often been based on a relatively limited geographic sampling coverage. Such local or regional sampling can be effective in detecting fine levels of population structure and revealing patterns of sympatric divergence but may mask other sources of substructuring, such as the presence of biogeographic or physical barriers to gene flow.

Here, we present the results of a microsatellite analysis of *An. gambiae* populations spanning the distribution of this species in the west of sub-Saharan Africa designed to assess geographic patterns of population structure within each molecular form.

## Materials and Methods

### Samples

**Figure 1:**
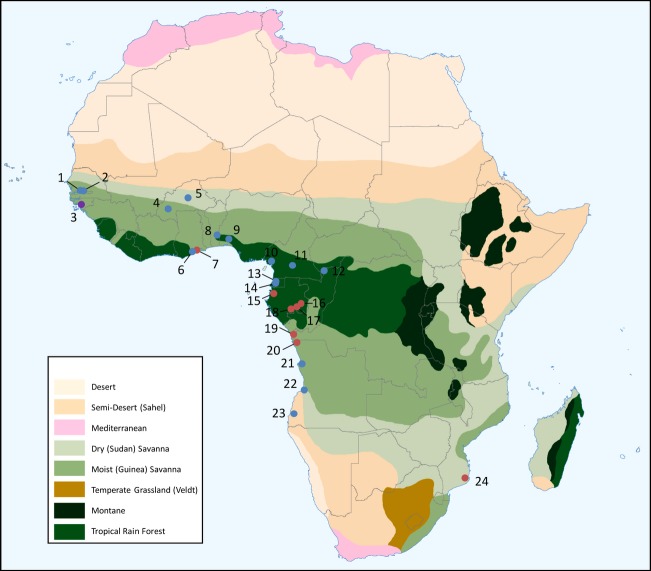
Map of Africa biomes (adapted from UNEP 2010) showing the location of the collection sites. Blue marks: M-form samples (identified by IGS-PCR); red marks: S-form samples; purple mark: locality with both M- and S-form samples. The Gambia: Wali Kunda (1), Maccarthy island (2); Guinea-Bissau: Bissau (3), Burkina Faso: Bobo-Dioulasso (4), Goundry (5); Ghana: Okyereko (6), Accra (7); Benin: Dassa (8); Nigeria: Kobape (9); Cameroon: Tiko (10), Simbok (11); Central African Republic (CAR): Bayanga (12); Equatorial Guinea: Ngonamanga (13), Bata (14); Gabon: Libreville (15), Benguia (16), Bakoumba (17), Dienga (18); Angola: Cabinda (19), Kikudo (20), Luanda (21), Cavaco (22), Namibe (23); Mozambique: Furvela (24). The dashed contour lines represent the approximate limits of the distribution of the S-form, and the dash-dotted contour line shows the limit of the distribution of the M-form, which is confined to west Africa.

Twenty-five *Anopheles gambiae* s.s. DNA samples were obtained from 24 collection sites in 12 African countries, between 1996 and 2006 (Fig. 1, see also Table S1 of the Supporting Information). These samples were collected mainly indoors by various adult sampling methods and identified to species by PCR (Scott et al. 1993), within the framework of previous entomological surveys. With the exception of a single site located in eastern Africa (Furvela, Mozambique), all sampling locations were in west Africa. The distribution of the west African sampling sites covered an overland distance of *ca*. 5700 km, from the Gambia to southern Angola.

Of the 25 samples analyzed, 16 were of the M-form and nine of the S-form according to the genotyping of the ribosomal DNA IGS marker (Favia et al. 1997; della Torre et al. 2001). The mean pair-wise distance among M-form sampling sites was 2031 km (median: 1810 km; SD: ± 1303 km) and 2129 km (median: 1977; SD: ± 1779 km) for S-form sampling sites. The mean pair-wise distance among S-form sites from west Africa (i.e. excluding the eastern African sample of Mozambique) was 1558 km (median: 797 km; SD: ± 1436 km). Although sympatric M- and S-forms are present in most west and central African sites, Bissau (Guinea-Bissau) was the only locality from which sympatric samples of both M- and S-forms were analyzed in this study. Information on sample size, year, type of collection, geographic coordinates, and biome type is given for each sample in Table S1 of the Supporting Information.

### Microsatellite genotyping

Thirteen microsatellite loci were genotyped. All loci were located on chromosome 3 to avoid potential bias resulting from reduced recombination or selective pressures acting at chromosomal inversions (frequent in chromosome 2) or linkage with genomic regions of M/S divergence on chromosome X (Lanzaro et al. 1998; Turner et al. 2005). Each locus was amplified individually by PCR with fluorescently labeled primers using the protocols described by Donnelly et al. (1999). Details of the microsatellites genotyped can be found in Table S1 of the Supporting Information.

### Microsatellite genotyping

Thirteen microsatellite loci were genotyped. All loci were located on chromosome 3 to avoid potential bias resulting from reduced recombination or selective pressures acting at chromosomal inversions (frequent in chromosome 2) or linkage with genomic regions of M/S divergence on chromosome X (Lanzaro et al. 1998; Turner et al. 2005). Each locus was amplified individually by PCR with fluorescently labeled primers using the protocols described by Donnelly et al. (1999). Details of the microsatellites genotyped can be found in Table S2 of the Supporting Information. Fragment analysis was performed by capillary electrophoresis on an automated sequencer (ABI®3730, Applied Biosystems, Foster City, CA, USA) at the Science Hill DNA Analysis Facility, Yale University. To control for variation in allele size scoring between capillary runs, the same positive controls, consisting of PCR products of two *An. gambiae* specimens from a laboratory colony, were used in all runs. One additional positive control (DNA template from a colony mosquito) and one negative control (no template) were also included to assess PCR quality. Allele sizes were scored from electropherograms using the software genemarker® (SoftGenetics, State College, PA, USA).

### Genetic data analysis

Genetic variation at each microsatellite locus was characterized by estimates of unbiased expected heterozygosity (Nei 1987) and allelic richness (El Mousadik and Petit 1996). The latter parameter was used to account for differences in sample sizes. Calculation of the estimates and comparisons among groups by permutation tests (1000 permutations) were performed using fstat v.2.9.3 (Goudet 1995). The same software was used to compute pair-wise estimates of the genetic differentiation parameter *F*_ST_ according to Weir and Cockerham (1984) and to assess their significance by permutation tests (1000 permutations). The number of shared alleles between groups was estimated in random subsamples of each group with size equal to the smallest group sample size. Exact tests against Hardy–Weinberg proportions and of linkage disequilibrium between pairs of loci were performed in genepop v.4.1 (Raymond and Rousset 1995). Presence of null alleles at each locus and sample was tested using the procedure implemented by microchecker with a 99% confidence interval (Van Oosterhout et al. 2004). The coalescent-based simulation approach implemented in lositan (Antao et al. 2008) was used to identify outlier microsatellites displaying unusually high or low *F*_ST_ values of by comparing observed *F*_ST_ estimates with values expected under neutrality (Beaumont and Nichols 1996). Runs were conducted under ‘neutral mean *F*_ST_’ and stepwise or infinite alleles mutation models using 50 000 simulations over all loci. The significance threshold for outlier detection was set at ≥0.95 percentile of simulations.

Bayesian clustering methods were used to detect population subdivision without *a priori* assumptions on population boundaries. Two types of clustering methods, namely spatial and nonspatial, were employed based on whether geographic information was included as a prior in the analysis. Spatial models generally perform better in cases of low differentiation (*F*_ST_ < 0.05) among populations (Chen et al. 2007).

The nonspatial Bayesian clustering analysis method implemented in structure 2.3.3 (Pritchard et al. 2000) was used to infer the number of genetic clusters (*K*) in the whole data set and within each molecular form separately. Analyses were carried out without prior information of sampling locations. A model with correlated allele frequencies within populations was assumed (*λ* = 1). The software was run with the option of admixture, allowing for some mixed ancestry within individuals, and the degree of admixture (α) was allowed to vary. For each value of *K* (*K* = 1–10), 10 independent runs were carried out with a burn-in period of 10 000 and 100 000 iterations. The Δ*K* statistic of Evanno et al. (2005) was calculated using structure harvester (Earl and vonHoldt 2012) to determine the most likely number of clusters. The information from the outputs of the 10 runs for each *K* was compiled by the greedy method implemented in clumpp (Jakobsson and Rosenberg 2007). Individual assignment to clusters was performed with a probability threshold (*T*_q_) determined by the analysis of simulated parental and admixed individuals generated by hybridlab v1.0 (Nielsen et al. 2006). From the initial whole-sample structure analysis, individuals showing a probability of membership *q*_i_ > 0.90 were selected to simulate 100 genotypes of each parental class and four hybrid classes (F1, F2, and backcrosses with each parental class). Simulated genotypes were analyzed by STRUCTURE under the same conditions as above. Following the example of Vähä and Primmer (2006), power and accuracy were calculated for four *T*_q_ values (0.70, 0.75, 0.80, and 0.90).

Spatial genetic clustering analysis was conducted with the whole data set and with M- and S-form data sets using the software tess v.2.3. (François et al. 2006; Chen et al. 2007). This method implements a Bayesian clustering algorithm that integrates genetic and spatial information to ascertain population structure without *a priori* population information, by inferring the most likely maximum number of clusters. As geographic coordinates were available only for each collection site, individual coordinates for each specimen were randomly generated within a circle with 10-km radius around the coordinate of each site. The 10-km radius was chosen based on previous observations on anopheline maximal flight distances that seem to vary around 9–12 km (Kaufmann and Briegel 2004). The two admixed models available in tess, car and bym (Chen et al. 2007; Durand et al. 2009), were used in the analysis. Ten independent runs were carried out with a burn-in period of 100 000 iterations and 100 000 replications for each value of *K*_*max*_ (*K* = 2–10). The Deviance Information Criterion (DIC) was used to select the admixture model that performed better and to infer the number of clusters. The maximum number of clusters was selected from DIC versus *K*_*max*_ plots as the lowest value at which the DIC curve reached a plateau. The estimated individual membership probabilities of the ten runs of the optimal *K*_*max*_ were averaged using the greedy algorithm in clumpp to correct for discrepancies between runs.

Principal coordinates analysis (PCoA) was used to visualize patterns of genetic differentiation among samples in a two-dimensional plot. Calculations were performed in genalex 6.41 (Peakall and Smouse 2006) using the standardized covariance method for the distance matrix conversion.

Isolation by distance was tested by the linear regression between logarithmic geographic distances and linearized 1/(1-*F*_ST_) values (Rousset 1997). Pair-wise overland distances between sites were estimated using the metric tool available in Google® Earth. The software genalex was used to assess the significance of the correlation by Mantel tests (1000 permutations).

Whenever multiple tests were performed, the nominal significance level (*α* = 0.05) was adjusted by the sequential Bonferroni procedure (Holm 1979).

## Results

A total of 967 *An. gambiae* were analyzed. Estimates of genetic diversity are shown in Table S3 of the Supporting Information. Mean allele richness (*R*_s_) of the microsatellite loci varied from 5.4 (AG3H242) to 12.7 (AG3H128) and expected heterozygosity (*H*_e_) from 0.575 (AG3H577) to 0.894 (AG3H128). There were 48 significant departures from Hardy–Weinberg proportions of 325 tests performed. These were associated with positive *F*_IS_ values indicating heterozygote deficits. Loci AG3H88, AG3H127, and AG3H750 comprised 39 (81.3%) of the 48 significant tests, suggesting that departures from Hardy–Weinberg expectations were locus-specific. Presence of null alleles was detected by microchecker in 44 of the 48 (92.7%) significant heterozygote deficits (Table S3, Supporting Information). There were 35 significant linkage disequilibrium (LD) tests of 1950 performed, of which 24 (68.6%) were observed in the sample from Cabinda, Angola (sample 19, Fig. 1) and 6 (17.1%) in Kobape, Nigeria (sample 9). Of the 13 loci analyzed for signatures of selection using lositan, only AG3H127 showed a significant signal of positive selection in both mutation models (Fig. S1, Supporting Information). Two additional loci, AG3H758 and AG3H93, displayed marginally significant signals of selection and only under the IAM or SMM mutation models, respectively.

**Figure 2:**
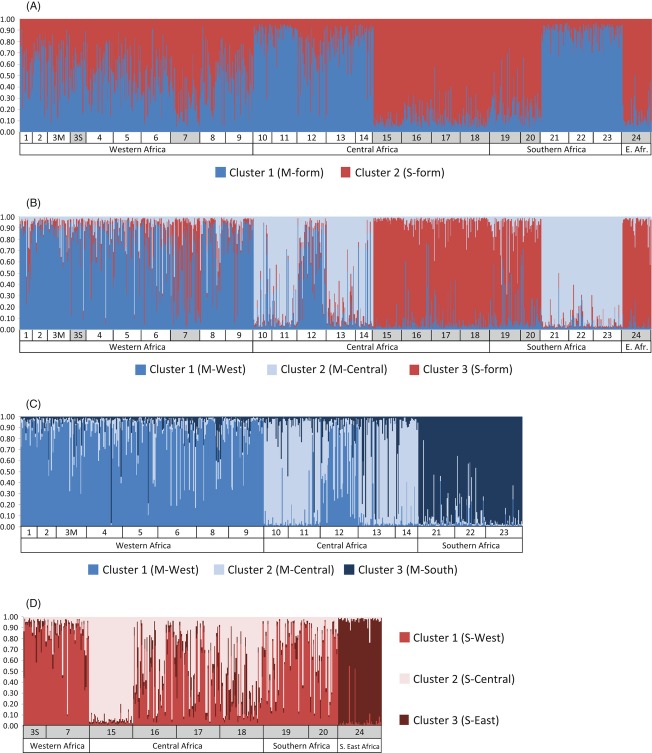
Bayesian clustering analysis implemented by structure (Pritchard et al. 2000). Localities are numbered according to Fig. 1 in a northwest–southeast direction along the *X*-axis (see also Table S1 of Supporting Information). White boxes indicate M-form and gray boxes indicate S-form samples as determined by the IGS marker. *Y*-axis: probability of ancestry to each cluster. In the graphs, each column corresponds to the multilocus genotype of a single individual partitioned into colors representing the probability of assignment to each cluster. (A) analysis performed with all samples (*N* = 967), *K* = 2; (B) analysis performed with all samples (*N* = 967), *K* = 3; (C) analysis performed with M-form samples only (*N* = 596), *K* = 3; (D) analysis performed with S-form samples only (*N* = 371), *K* = 3.

The results of the Bayesian clustering analysis implemented in structure are shown in Fig. 2. Graphical representations of Evanno's Δ*K* can be seen in Fig. S2 of the Supporting Information. When all samples were analyzed together, the optimum number of clusters was *K* = 2. This partitioning generally corresponded to the M (cluster 1) and S (cluster 2) molecular form composition of the samples and it was independent of geographic location. However, samples from west African sites (i.e. samples 1–9 in Fig. 2, *K* = 2) displayed more inconsistencies between the form determined by the IGS marker and the respective genetic background when compared to samples from central and southern Africa. In west African samples, the average probability of assignment to cluster 1 for M-form specimens was 0.515 and 0.636 for S-form assignment to cluster 2. When individuals were assigned to each cluster based on a *T*_q_ ≥ 0.8, as determined by the analysis of simulated data (see Table S4, Supporting Information), there were only 8.7% (25 of 289) M-form individuals assigned to cluster 1 and 33.3% (23 of 69) S-form individuals to cluster 2. The proportion of individuals with admixed ancestry (i.e. 0.20 < *T*_q_ < 0.80) was 83.7% and 65.2% for M- and S-form, respectively. In contrast, the average probabilities of assignment for M- and S-form individuals from central and southern African sites were 0.831 and 0.847, respectively. The proportion of consistent assignments was also much higher: 73.3% (225 of 307) in the M-form and 75.8% (229 of 302) in the S-form. The second highest Δ*K* value corresponded to *K* = 3. Here, M-form populations were subdivided into two genetic clusters (Fig. 2, *K* = 3): cluster 1 contained mainly individuals from the samples collected in west Africa (samples 1–9) and also from Bayanga, CAR (sample 12); cluster 2 included the remaining samples from central Africa (samples 10, 11, 13, and 14) and Angola (samples 21–23). These results did not differ qualitatively when analyses were repeated excluding the three loci that revealed most heterozygote deficits indicating that locus-specific Hardy–Weinberg deficits had little impact in the analysis (Fig. S3, Supporting Information).

structure was also performed within each molecular form separately. When M-form samples were analyzed, a third subdivision was evident (Fig. 2, M-form). West African samples were again grouped in a single cluster (cluster 1, samples 1-9, 12), but there was a separation between central African samples (cluster 2, samples 10-11, 13-14) and the southernmost samples from Angola (cluster 3, samples 21-23). The only exception to this geographic partitioning was the sample from Bayanga (sample 12). This sample has a central African location, but individuals displayed a higher probability of assignment to cluster 1 (mean = 0.633) compared to cluster 2 (mean = 0.289). For *T*_q_ ≥ 0.80, 46.7% of the 45 individuals analyzed were assigned to the west African cluster 1 and only 2 individuals (4.4%) were assigned to the central African cluster 2.

Subdivision among S-form populations was also observed when structure analysis was performed with these samples only (Fig. 2, S-form). The two west African samples (Bissau, 3S and Accra, 7) were grouped into a west African cluster (cluster 1). In central Africa, a second cluster was detected (cluster 2). This genetic background predominates in the sample from Libreville, Gabon (sample 15) and gradually intergrades southwards with cluster 1. The proportion of individuals assigned to cluster 2 (*T*_q_ ≥ 0.80) decreased from 97.8% in Libreville (sample 15) to 28.9% (Dienga, 18), 17.8% (Benguia, 16), 13.3% (Bakoumba, 17), 8.5% (Cabinda, 19), and 6.7% in the southernmost Kikudo (sample 20). Finally, a third cluster comprised specimens from the southeast African sample of Furvela (sample 24), in Mozambique.

**Figure 3:**
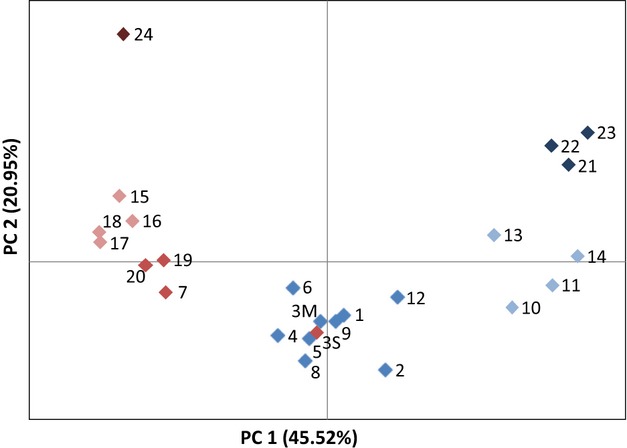
Principal coordinates analysis of the 25 *An. gambiae* samples. Each mark represents a sample numbered according to Fig. 1. Marks are colored according to the within-form genetic clusters revealed by structure (Fig. 2). Blue: M-west, light blue: M-central, dark blue: M-south; red: S-west, light red: S-central, dark red: S-east.

The geographic structuring of M-form populations was also evident in the principal coordinates analysis (Fig. 3). The distribution of the M-form samples in the plot reflects their geographic grouping into west, central, and southern clusters. The S-form samples were clearly separated from the M-form with the single exception of Bissau (3S, Fig. 3). The separation between west and central African S-form samples was less pronounced than in the M-form. The S-form sample of Furvela, Mozambique (sample 24), was placed as an outlier of the S-form group, in agreement with the results obtained by structure.

**Figure 4:**
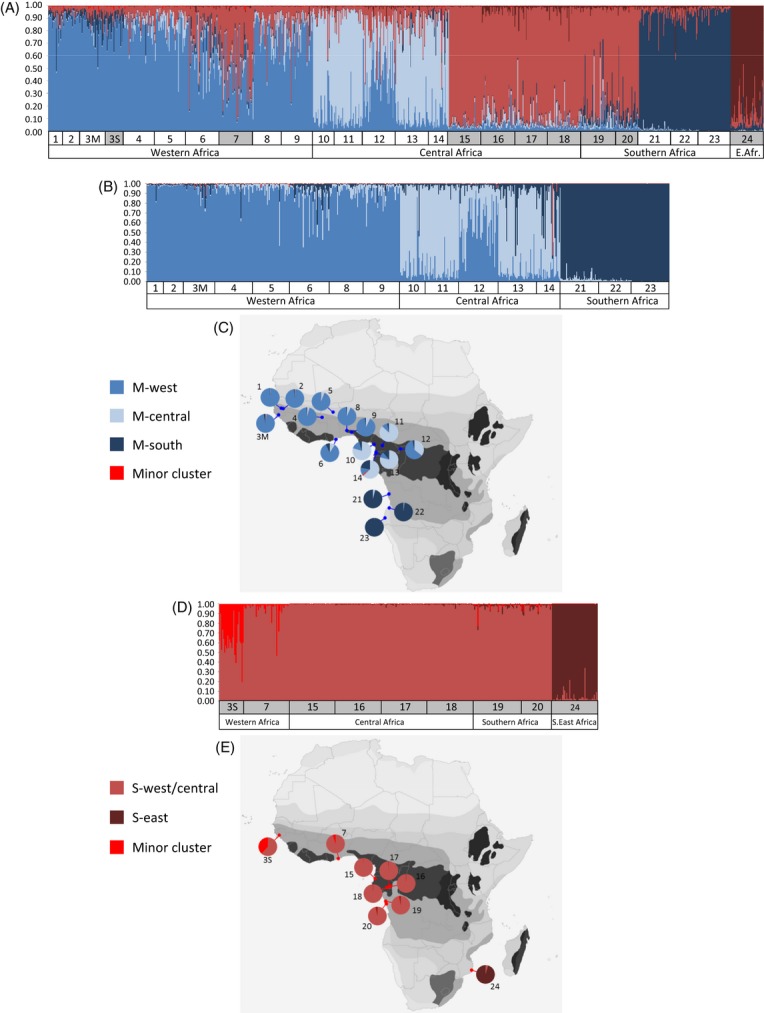
Individual assignment plots and maps showing mean membership probabilities to each cluster at each locality, obtained by tess (Chen et al. 2007). The bar plots depict individual assignment probabilities averaged for the ten runs using clumpp (Jakobsson and Rosenberg 2007). The maps show pie charts of the average probability of membership to each cluster for each locality. Samples are numbered according to Fig. 1 and Table S1 (Supporting Information). (A) analysis performed with all samples (i.e. both M- and S-form), with clusters colored according to the labels of the following bar plots; (B and C) analysis performed with M-form samples only; (D and E) analysis performed with S-form samples only.

The results of the spatially explicit analysis conducted in tess were very similar for the two admixture models used. The car model gave, however, less-dispersed DIC values between runs, so that only the results for this model are presented (Fig. S4, Supporting information). When both M- and S-form samples were analyzed together, an optimal *K*_*max*_ = 6 was obtained (Fig. 4, A). There were three major clusters that consisted in the partitioning of the M-form into west, central, and south clusters, thus confirming the results of the nonspatial analyses. In the S-form, however, only the east African sample of Furvela, Mozambique (sample 24), formed a distinct cluster, whereas the remaining S-form samples from west and central Africa grouped together. There was one additional minor cluster in which the highest individual probability of membership was only 0.38, for a specimen from Bata (sample 14). The spatial analysis of M- and S-form samples alone did not disclose any additional substructuring. For the M-form, a *K*_*max*_ = 4 was obtained confirming west, central, and southern clusters (Fig. 4, B and C). A fourth minor cluster comprised again the same single individual from Bata, Equatorial Guinea (sample 14) with a probability of membership *q*_i_ = 0.731. The assignment of this individual into a minor cluster was also consistent in the tess analyses performed with 10 loci (i.e. excluding the three loci with greatest heterozygote deficits; Fig. S3, C and D). This consistency led us to re-analyze the IGS molecular identification (Scott et al. 1993) of this and the other specimens of this locality. This revealed the presence of two misidentified individuals. One was found to be *Anopheles melas*, another sibling species of the *An. gambiae* complex, and corresponded to the individual assigned to the minor cluster. The other gave a banding pattern consistent with a hybrid between *An. melas* and *An. gambiae s.s*. This individual was assigned to the M-west cluster with *q*_i_ = 0.621. Removing these two individuals had little influence on the estimates of pair-wise genetic differentiation between this locality and the others (Table S6). For the S-form, an optimal *K*_*max*_ = 3 also confirmed the separation of the east African sample of Furvela, Mozambique (sample 24), but did not disclose any subdivision between central and west African samples (Fig. 4, D and E). There was one additional minor cluster represented by five specimens, four from Bissau (sample 3S) and one from Accra (sample 7).

**Figure 5:**
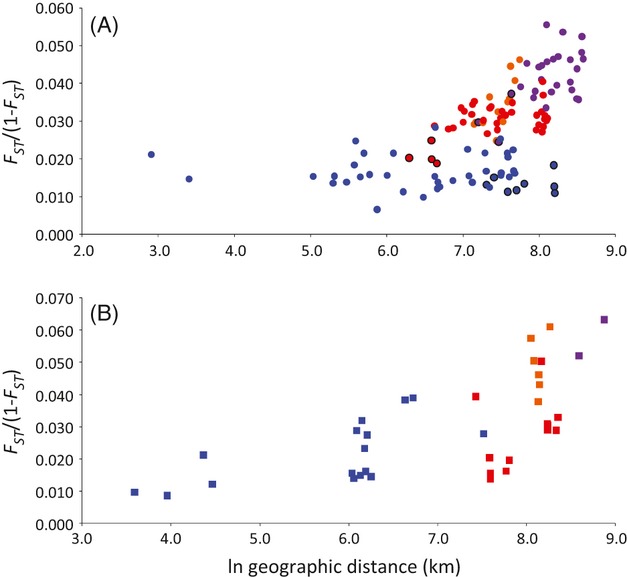
Plots of the regression between *F*_ST_/(1-*F*_ST_) and logarithmic geographic distance. (A) M-form. Blue circles: comparisons between localities for which the majority of individuals were assigned to the same genetic cluster (i.e. M-west, M-central, and M-south). Red circles: comparisons between M-west and M-central localities. Orange circles: comparisons between M-central and M-south localities. Purple: comparisons between M-west and M-south localities. Circles with a black line correspond to comparisons involving the locality of Bayanga (CAR), which was considered as representative of the M-west cluster. (B) S-form. Blue squares: comparisons between localities belonging to the same genetic cluster (i.e. S-west, S-central, and S-east/Mozambique). Red squares: comparisons between S-west and S-central localities. Orange squares: comparisons between S-central localities and S-east/Mozambique. Purple squares: comparisons between S-west localities and S-east/Mozambique.

Significant positive slopes were obtained for all the regressions of (*F*_ST_/1-*F*_ST_) with logarithmic distance (Table S5, Supporting Information). The proportion of the variation explained by the regression (*r*^2^) was generally low, particularly when both M- and S-form were analyzed together (all samples, Table S5, Supporting Information). The largest *r*^2^ value was recorded for the regression involving S-form samples (0.43). When the most distant S-form sample of Furvela, Mozambique (sample 24), was removed, the regression remained significant but with a lower *r*^2^ (0.28). Plots of the regression of linearized *F*_ST_ and logarithmic distance for M- and S-forms are shown in Fig. 5. For the M-form, comparisons between sampling sites within the same genetic cluster (obtained by structure) had in general lower *F*_ST_ than comparisons involving sites from distinct genetic clusters (Fig. 5, A). The mean of pair-wise *F*_ST_ estimates between samples within each cluster varied between 0.015 and 0.022, corresponding to comparisons between collection sites 18–3658 km apart (Table S6, Supporting Information). The mean of pair-wise *F*_ST_ between samples from different clusters ranged from 0.030 to 0.042 and involved comparisons with distances between 541 km and 5317 km. This pattern was not so evident in the S-form, where differentiation appears to reflect less cluster ancestry and depend more on geographic distance (Fig. 5, B).

Estimates of genetic diversity for each genetic cluster within the M-form and for the S-form are shown in Table 1. There was a trend for a south–north increase in diversity within the M-form. Estimates of *R*_s_ and *H*_e_ were lowest in the M-south cluster, intermediate in the M-central, and highest in the M-west cluster. These differences were significant for both parameters (permutation tests, *R*_s_: *P* = 0.001; *H*_e_: *P* = 0.027). The S-form displayed *R*_s_ and *H*_e_ values similar to those of the M-central cluster. The average number of shared alleles among clusters was higher between the S-form and M-west clusters than between any other comparison (Table 1). These two clusters also had the lowest pair-wise *F*_ST_ estimate (0.023) with the highest (0.075) being observed between S-form and M-south (Table 1).

**Table 1:** Estimates of genetic diversity, pair-wise *F*_ST_, and proportions of shared alleles among S-form and M-form clusters

	M-west	M-central	M-south	S-form
*R*_*s*_	8.8 (0.3)	7.5 (0.2)	6.3 (0.4)	7.4 (1.0)
*H*_*e*_	0.806 (0.016)	0.773 (0.013)	0.720 (0.027)	0.743 (0.051)
M-west	–	0.030	0.048	0.023
M-central	11.5	–	0.035	0.061
M-south	9.4	8.7	–	0.075
S-form	13.0	10.8	8.9	–

*H*_e_, mean over loci expected heterozygosity; *R*_*s*_, mean over loci allele richness; in parenthesis: standard deviation of mean; above diagonal: F_*ST*_ estimates (all significant, *P* < 0.001); below diagonal: mean over loci number of shared alleles estimated in randomly selected subsamples of each group with samples size equal to the lowest sample size (M-south, *N* = 124).

## Discussion

The macrogeographic scale microsatellite analysis on *An. gambiae* presented here revealed a significant association between genetic differentiation and geographic distance. This pattern of isolation by distance was not an unexpected result given the relatively low active dispersal capacity of this mosquito (<13 km, Kaufmann and Briegel 2004) and also agrees with a previous study covering similar geographic ranges (Lehmann et al. 2003). However, isolation by distance appears not to be the only factor shaping the genetic structure of this species. Two additional sources of variation were disclosed: the well-known subdivision of the species into the M and S molecular forms and the split of the M-form into three geographic clusters corresponding to west, central, and southern African populations.

Subdivision corresponding to the two molecular forms was revealed by both Bayesian clustering analyses and was also confirmed by PCoA. This pattern was detected using molecular markers located outside the previously described genomic regions of divergence (Turner et al. 2005; White et al. 2010). It was also independent of the geographic location. At *K* = 2 of the structure analysis, all M-form samples clustered together regardless of being from west, central, or southern Africa. Likewise, the S-form samples from west and central Africa also clustered with the East African sample of Mozambique. The single exception was the clustering of the majority of the S-form individuals from Bissau in the M-form cluster, which reflects the high levels of inter-form hybridization and asymmetric introgression previously described for this region (Oliveira et al. 2008; Caputo et al. 2011; Marsden et al. 2011). The introgression of more M-form genes into the S-form detected in these reports agrees with the position of the S-form sample from Bissau in the west M-form cluster of the PCoA conducted in this study.

The degree of inter-form differentiation appeared to be higher in central and southern African samples than in west African ones, judging by the individual probabilities of assignment to M- and S-form clusters obtained in structure at *K* = 2. An explanation for this observation could be the nearly monotypic composition of *An. gambiae* in some of the collection sites. This is the case for S-form samples of Mozambique, Gabon and northern Angola and also for the M-form samples of Angola (Pinto et al. 2002; Calzetta et al. 2008). However, this hypothesis is less probable for the sites sampled in Cameroon and Equatorial Guinea, in which both forms have been found in sympatry at minimum relative frequencies of *ca*. 10:90 (Moreno et al. 2007; Ridl et al. 2008; Simard et al. 2009; Weetman et al. 2010; Kamdem et al. 2012).

There is evidence that inter-form gene flow and introgression varies across the *An. gambiae* distribution range. In the central African region, the degree of inter-form divergence appears to be highest and coincident with no reported MS hybrids (della Torre et al. 2005; Simard et al. 2009), although there is evidence for at least sporadic recent gene flow (Etang et al. 2009; Weetman et al. 2012). In contrast, the isolation between forms seems to be less marked in west Africa. Here, MS hybrid rates have been found to vary greatly, from ∼1% (della Torre et al. 2005; Costantini et al. 2009) to over 20% (Caputo et al. 2008; Oliveira et al. 2008). High levels of inter-form hybridization and a pattern of asymmetric introgression have been described in Guinea-Bissau (Oliveira et al. 2008; Caputo et al. 2011; Marsden et al. 2011). Low inter-form differentiation was also reported in a previous microsatellite analysis of samples from different ecological zones in Ghana (Yawson et al. 2007). These results contrast with the high levels of inter-form differentiation revealed by genome-wide SNP analyses in *An. gambiae* from Ghana (Weetman et al. 2010) and also from Mali (Neafsey et al. 2010). This discrepancy might be influenced by the propensity of microsatellites to underestimate genetic differentiation as a result of allelic homoplasy (Estoup et al. 2002). However, in the SNP analyses of M- and S-forms from Ghana, differentiation was markedly heterogeneous and far lower on chromosome-3 than on chromosome-2 and chromosome X (Weetman et al. 2012). Thus, differences might also be explained by variation in the genomic location of markers. Comparative genome-wide SNP analysis of samples from central and west African regions displaying varying levels of hybridization showed that the degree of genomic divergence was dependent on the amount of realized gene flow between forms (Weetman et al. 2012). Altogether, these results point to a considerable variation in the degree of isolation between molecular forms throughout the species range. This variation may be a consequence of an intricate assemblage of factors such as local or regional differences in the stage or history of the speciation process, the occurrence of secondary contact zones, and differences in the ecological trade-offs of hybridization (Caputo et al. 2011; Marsden et al. 2011).

Additional partitioning into three distinct geographic M-form clusters corresponding to west, central, and southern African populations was revealed by both spatial and nonspatial Bayesian clustering analyses and also by PCoA. This subdivision appears to be coincident with the transition from a rainforest biome to northern and southern savannah biomes, respectively. The genetic discontinuity imposed by the forest-savannah transition is not complete, as evidenced by the maintenance of a significant isolation-by-distance signal across all M-form samples and also by the presence of a locality (Bayanga, CAR) displaying a higher proportion of an M-west genetic background in spite of its rainforest location. Bayesian clustering methods may overestimate genetic structure by generating spurious clusters when applied to populations displaying isolation by distance (Frantz et al. 2009; Schwartz and Mckelvey 2009). However, pair-wise *F*_ST_ values within clusters were generally lower than those involving comparisons between clusters, even when the distances between sampling sites of the same genetic cluster were similar to those between sampling sites of different clusters (Fig. 5, A). This suggests that differentiation within the M-form is not only dependent on geographic distance but that restrictions to gene flow may also be present. The intermediate *F*_ST_ values of the sample of Bayanga in the plot of Fig. 5 (A) are also consistent with a higher admixture between two distinct genetic clusters in this particular locality. Also, levels of differentiation do not seem to have been influenced by temporal differences between samples. Nonsignificant *F*_ST_ values were obtained between samples from the Gambia and Guinea-Bissau, located *ca*. 200 km apart, in spite of a 7-year interval between these collections.

Initial evidence of a separation between west and central African M-form *An. gambiae* populations emerged from two previous microsatellite-based studies. In the only microsatellite-based continent-wide study carried out before the present one, the grouping of Senegal and Ghana samples apart from central African ones was observed in a *F*_ST_-based neighbor-joining population tree (Lehmann et al. 2003). Moreover, a high degree of genetic differentiation was found between M-form populations from a savannah area in Mali and those from a forested area in Cameroon, suggesting that M-form may not be a single entity (Slotman et al. 2007).

Population subdivision associated with forest-savannah transitions is not uncommon. A similar scenario was recently described within the *An. gambiae* sibling species *Anopheles melas* Theobald, in which genetically distinct west and central/southern African clusters were detected with a degree of divergence comparable to that observed among other species of the *An. gambiae* complex (Deitz et al. 2012). Significant differentiation between rainforest populations and one southern savannah population of *Anopheles nili* (Theobald) in central Africa also suggested a role of the evergreen forest as a barrier to gene flow in this vector species (Ndo et al. 2010). A recent study has also shown the occurrence of a cryptic central African group genetically distinct from west African populations within the tsetse fly *Glossina palpalis palpalis* Robineau-Desvoidy (Dyer et al. 2009). Altogether, these results are consistent with a role of the transition between rain forest and savannah biomes as a barrier to gene flow in insect species.

Recent studies have shown that central African M-form populations are becoming more adapted to densely urbanized areas where they explore polluted breeding sites of anthropogenic nature (Simard et al. 2009; Kamdem et al. 2012). In contrast, west African M-form populations appear more closely associated with irrigated agricultural areas, occupying more permanent breeding sites such as rice fields and irrigation reservoirs (Gimonneau et al. 2012). Local adaptation to different ecological niches coupled with the effect of isolation by distance and restrictions to mosquito active dispersal imposed by the rainforest environment could explain the observed patterns of population subdivision within the M-form.

Another factor that may have contributed to the differentiation between west and central African M-form clusters could be a higher degree of genetic introgression between M- and S-forms in west Africa. This effect is suggested by the highest mean number of shared alleles and lowest pair-wise *F*_ST_ estimate between the M-west cluster and the S-form, in line with a hypothesis of highest introgression between these clusters. Introgression may also explain the higher levels of genetic diversity of the M-west cluster as measured by estimates of *H*_e_ and *R*_s_. The data suggest that substantial MS inter-form introgression is a less-probable cause for the differentiation between central and southern M-form samples, because evidence of inter-form gene flow (i.e. admixture in the *K* = 2 analysis of structure) was much lower in these samples. However, sequence analysis of an X-linked locus revealed that the majority of M-form individuals in Angola had a 16-bp insertion that was fixed in the S-form but absent in M-form individuals from west and central Africa (Choi and Townson 2012), a finding that suggests inter-form introgression has occurred in this geographic region.

The results obtained for the S-form did not conclusively show a genetic discontinuity at the transition between rainforest and savannah. A central African S-form cluster was detected by structure analysis but appears to be mostly represented by a single sample (Libreville). Rainforest samples of Gabon also appeared more closely related in the PCoA. However, S-form samples from savannah biomes in west Africa (Ghana) and Angola were grouped together in the PCoA and into a single cluster in structure. The intergradation between S-form clusters observed southwards of Libreville in the STRUCTURE analysis also suggests gradual differentiation, in line with an expectation of isolation by distance. Moreover, the results of the spatial genetic analysis conducted by tess for the S-form did not show a clustering of central African samples within the rainforest belt. Instead, the two major clusters corresponded to the separation of the East African sample of Mozambique from central and west African samples. However, it should be noted that in spite of the continent-wide distribution of the S-form, the number of samples available for this study was quite limited, particularly in west Africa. Moreover, central African samples were also concentrated within a relatively small area separated by a maximum distance of <500 km. This restricted sampling could have influenced the results, especially for the spatial cluster analysis as the accuracy of these methods tends to increase with the inclusion of more spatial points (Guillot et al. 2009). Thus, while our data suggest that isolation by distance may be the predominant force in genetic structuring of the S-form, greater geographic coverage would be required to confirm if a pattern of population subdivision associated with the forest-savanna transition also occurs in this form. The third minor cluster detected included only five specimens, four of which were collected in Bissau. While this minor cluster may represent an artifact of the analysis, as the effective number of clusters may be lower than *K*_*max*_ (Durand et al. 2009), it may also represent admixed individuals between M- and S-forms, given the high levels of hybridization reported for this locality (Oliveira et al. 2008; Caputo et al. 2011; Marsden et al. 2011). In fact, this particular S-form sample from Bissau grouped together with the west African M-form samples in the PCoA and was not distinguishable from the M-form in both spatial and nonspatial Bayesian analyses performed with all samples. The differences found between spatial and nonspatial clustering models in the S-form highlight the importance of adding a spatial component into the analysis especially in cases where isolation by distance is likely to influence the patterns of population differentiation. When all samples were analyzed together by tess, the optimal *K* obtained reflected both the M/S subdivision and the geographic partitioning within each form.

The apparent shallow differentiation between west and southern African S-form samples is consistent with previous studies pointing to an overall shallow population differentiation within this form (Lehmann et al. 1999, 2003). These studies have detected a single major subdivision of S-form populations in east Africa associated with gene-flow restrictions imposed by the rift valley. In contrast with the M-form, whose distribution is limited to the occidental side of Africa, the relatively continuous distribution of the S-form throughout the sub-Saharan continent may provide a connection between west and southern S-form populations through the intermediate central African region west to the Rift Valley. On the other hand, the heterogeneous haplotype distribution of genes conferring knockdown insecticide resistance is consistent with a possible partitioning between rainforest and savannah S-form populations (Pinto et al. 2007; Lynd et al. 2010). While differences in insecticide selection pressure are likely to be the major force shaping the distribution of *kdr* haplotypes, forest/savannah restrictions to gene flow may also contribute to the observed heterogeneities.

The results obtained in this study show that in addition to the M- and S-forms partitioning and to the existence of local or regional genetic variants (Coluzzi et al. 1979; Riehle et al. 2011), population subdivision may occur at a macrogeographic scale in *An. gambiae*, at least within the M-form. This trend appears to be associated with the transition between forest and savannah biomes and appears to be evident both northwards and southwards from the central African rainforest belt. This complexity is of importance to the management of malaria vector control programs. A genetic discontinuity between savannah and forest biomes is likely to influence dispersal and distribution of genes of practical importance to malaria epidemiology and control, such as genes associated with insecticide resistance or with vector competence.

## Data archiving statement

Data for this study available on Dryad: doi:10.5061/dryad.201rm.

## Supporting Information

**Table S1.** Geographic information, year of collection and molecular form composition of the samples analyzed.

**Table S2.** Microsatellite loci genotyped.

**Table S3.** Microsatellite genetic diversity estimates according to collection site.

**Table S4.** Power and accuracy of the Bayesian clustering analysis implemented by structure (Pritchard et al. 2000) to detect M and S form simulated individuals (*N* = 100 for each form).

**Table S5.** Isolation by distance model regressions of *F*_ST_/(1-*F*_ST_) on logarithm of distance.

**Table S6.** Pair-wise estimates of *F*_ST_ (below diagonal) and geographic distance (above diagonal, in kilometres) between sampling sites.

**Figure S1.** Plots between genetic differentiation and expected heterozygosity to detect candidate microsatellite loci under selection according to the method implemented in lositant (Antao et al. 2008

**Figure S2.** Graphics of Evanno's *ΔK* for the different Bayesian clustering analyses implemented by structure.

**Figure S3.** Bayesian clustering analysis implemented by structure (Pritchard et al. 2000) and spatially explicit analyses implemented by tess (Chen et al. 2007), performed with 10 microsatellite loci.

**Figure S4.** Plots of DIC values (*Y*-axis) against *K*_*max*_ (*X*-axis) obtained for tess analysis (Chen et al. 2007) under two admixture models (car and bym).
