# Supplementary material for: Geographic population structure of the African malaria vector Anopheles gambiae suggests a role for the forest-savannah biome transition as a barrier to gene flow
Source: Evol Appl. 2013 Jun 10;6(6):910–24. doi: 10.1111/eva.12075 (PMC3779092; doi:10.1111/eva.12075)
Supplement: Supplementary file 1 [file eva0006-0910-SD1.docx]

Table S1. Geographic information, year of collection and molecular form composition of the samples analyzed

| Region | Country | Nº | Locality | Latitude | Longitude | Biome | Year | Collection method | Form | *N* |
| --- | --- | --- | --- | --- | --- | --- | --- | --- | --- | --- |
| western Africa | The Gambia | 1 | Wali Kunda | 13.5732 | -14.9235 | Msav | 2003 | IR | M | 19 |
|  |  | 2 | Maccarthy island | 13.5383 | -14.7668 | Msav | 2003 | IR | M | 23 |
|  | Guinea Bissau | 3M | Bissau | 11.8911 | -15.5820 | Msav | 1996 | IR | M | 36 |
|  |  | 3S |  |  |  |  |  | IR | S | 24 |
|  | Burkina Faso | 4 | Bobo-Dioulasso | 11.2010 | -4.3092 | Msav | 2001 | IR | M | 42 |
|  |  | 5 | Goundry | 12.5149 | -1.3372 | Dsav | 2000 | IR | M | 43 |
|  | Ghana | 6 | Okyereko | 5.4145 | -0.6045 | Msav | 2006 | IR | M | 45 |
|  |  | 7 | Accra | 5.5441 | -0.2386 | Msav | 2006 | IR | S | 45 |
|  | Benin | 8 | Dassa | 7.7794 | 2.1884 | Msav | 2003 | IR | M | 39 |
|  | Nigeria | 9 | Kobape | 7.3115 | 3.4971 | Msav | 2001 | LT | M | 42 |
| central Africa | Cameroon | 10 | Tiko | 4.0884 | 9.3505 | Rfor | 2003 | IR | M | 29 |
|  |  | 11 | Simbok | 3.8186 | 11.4710 | Rfor | 1999 | IR | M | 38 |
|  | C.A.R. | 12 | Bayanga | 2.9175 | 16.2579 | Rfor | 2003 | IR | M | 45 |
|  | Equatorial Guinea | 13 | Ngonamanga | 2.1372 | 9.7915 | Rfor | 2004 | IR | M | 45 |
|  |  | 14 | Bata | 1.8723 | 9.7919 | Rfor | 2004 | IR | M | 26 |
|  | Gabon | 15 | Libreville | 0.3844 | 9.4546 | Rfor | 2000 | LC | S | 45 |
|  |  | 16 | Benguia | -1.6258 | 13.4411 | Rfor | 1999 | LC | S | 45 |
|  |  | 17 | Bakoumba | -1.8269 | 13.0166 | Rfor | 2000 | LC | S | 45 |
|  |  | 18 | Dienga | -1.8470 | 12.6920 | Rfor | 1999 | LC | S | 45 |
| southern Africa | Angola | 19 | Cabinda | -5.5760 | 12.1852 | Msav | 2003 | IR | S | 47 |
|  |  | 20 | Kikudo | -6.1488 | 12.3708 | Msav | 2002 | IR | S | 30 |
|  |  | 21 | Luanda | -8.8214 | 13.2911 | Msav | 2002 | IR | M | 44 |
|  |  | 22 | Cavaco | -12.5669 | 13.4119 | Dsav | 2005 | IR | M | 37 |
|  |  | 23 | Namibe | -15.1987 | 12.1557 | Sdes | 2002 | IR | M | 43 |
| E. Afr. | Mozambique | 24 | Furvela | -23.7162 | 35.2986 | Dsav | 2004 | LT | S | 45 |

E. Afr.: east Africa. CAR: central African Republic. Geographic coordinates of each sampling site were obtained either by on site Global Positioning System measurement or from Google Maps©. Latitude and longitude are in decimal degrees. Msav: moist (Guinea) savanna, Dsav: dry (Sudan) savanna, Rfor: tropical rain forest, Sdes: semi-desert (Sahel). Year: year of sample collection. Form: IGS-based molecular form of *An. gambiae*. *N*: sample size. Collection method: IR: indoor resting collections, LC: landing captures, LT: CDC light traps.

Table S2. Microsatellite loci genotyped

| Locus | Location | Repeat | Dye | Primers | *T_A_* |
| --- | --- | --- | --- | --- | --- |
| AG3H93 | 3R (29A) | (GT)_4+7_ | ned | *f-*TCC CCA GCT CAC CCT TCA AG  *r-*GGT TGC ATG TTT GGA TAG CG | 54 |
| AG3H128 | 3R (29C) | (GT)_21_ | 6-fam | *f-* CGG GAC GGC TAG ATA AAG CG  *r-* CCG GGC GAC ATA ACC CAC CC | 56 |
| AG3H59 | 3R (29D) | (GT)_9_ | hex | *f-* CCC CTA TTA AAC CCT GGA CG  *r-* TGT TGT TGC CCT GCG TTA CC | 54 |
| AG3H249 | 3R (30D) | (GT)_15_ | ned | *f-* ATG TTC CGC ACT TCC GAC AC  *r-* GCG AGC TAC AAC AAT GGA GC | 54 |
| AG3H119 | 3R (31B) | (GT)_6_ | hex | *f-* GGT TGA TGC TGA AGA GTG GG  *r-* ATG CCA GCG GAT ACG ATT CG | 54 |
| AG3H555 | 3R (32C) | (GT)_8_ | ned | *f-* GCA GAG ACA CTT TCC GAA AC  *r-* TGT CAA CCC ACA TTT TGC GC | 54 |
| AG3H88 | 3R (34B) | (GT)_9_ | 6-fam | *f-*TGC GGC GGT AAA GCA TCA AC  *r-*CCG GTA ACA CTG CGC CGA C | 56 |
| AG3H127 | 3L (39A) | (GT)_12_ | hex | *f-* CCT CTA CCT CGA TTA CCG TG  *r-* GTC AGG CAA TTG GAA AGA GC | 54 |
| AG3H750 | 3L (41C) | (GT)_8_ | ned | *f-* gca aaa aag ctt ctc ccc  *r-* tta gct acc gtc gac gct tc | 54 |
| AG3H577 | 3L (42A) | (GT)_16_ | 6-fam | *f-* TTC AGC TTC AGG TTG GTC TC  *r-* GGG TTT TTT GGC TGC GAC TG | 56 |
| AG3H758 | 3L (43A) | (GT)_11_ | 6-fam | *f-*TGA TTT GCC AGT TCT GCC AG  *r-*GTG ATT GGA GTG GCT AGT GG | 54 |
| AG3H242 | 3L (45B) | (GT)_8_ | hex | *f-*TTC ATT TCC ACC GCA GCT GC  *r-* GGC GAC ACT CAA TCC TTC C | 56 |
| 45C1 | 3L (45C) | (TG)_4+7+4_ | hex | *r-*AAA AGT GGT GAC CGA GTG AC  *f-*ATC TTC AAC ACT TCA GCA CG | 54 |

Location: physical location in chromosome 3 (arm L or R and division). Repeat: Type of repeat and size of cloned fragment (Zheng *et al.*, 1996. *Genetics* 143: 941-952; 45C1: Wang *et al.*, 1999. *Parasitology Today* 15: 33-37). Dye: fluorescent label in forward primer. Primers: *f-*forward and *r*-reverse. *T_A_*: annealing temperature (°C).

Table S3. Microsatellite genetic diversity estimates according to collection site

| Site |  | H242 | H128 | H249 | H119 | H555 | H127 | H577 | H750 | H59 | H88 | H758 | H93 | 45C | Mean |
| --- | --- | --- | --- | --- | --- | --- | --- | --- | --- | --- | --- | --- | --- | --- | --- |
| Wali Kunda (1) | *R_s_* | 7 | 14 | 11 | 11 | 10 | 8 | 5 | 10 | 5 | 9 | 10 | 10 | 7 | 9 |
|  | *H_e_* | 0.734 | 0.925 | 0.874 | 0.890 | 0.846 | 0.877* | 0.588 | 0.891 | 0.753 | 0.861 | 0.898* | 0.868 | 0.827 | 0.833 |
|  | *F_IS_* | 0.000 | 0.000 | 0.157 | 0.000 | 0.005 | 0.463 | 0.000 | 0.064 | 0.115 | 0.226 | 0.296 | 0.030 | 0.000 | 0.104 |
| McCarthy island (2) | *R_s_* | 4 | 14 | 12 | 9 | 9 | 7 | 8 | 11 | 7 | 9 | 11 | 9 | 8 | 9 |
|  | *H_e_* | 0.479 | 0.934 | 0.873 | 0.861 | 0.861 | **0.881*** | 0.741 | 0.881 | 0.841 | 0.845 | 0.907 | 0.778 | 0.866 | 0.827 |
|  | *F_IS_* | 0.000 | 0.027 | 0.103 | 0.050 | 0.040 | 0.849 | 0.000 | 0.161 | 0.000 | 0.190 | 0.040 | 0.050 | 0.247 | 0.135 |
| Bissau M-form (3M) | *R_s_* | 6 | 14 | 12 | 8 | 8 | 8 | 8 | 8 | 7 | 9 | 11 | 10 | 8 | 9 |
|  | *H_e_* | 0.617 | 0.925 | 0.877 | 0.849 | 0.838 | **0.804*** | 0.731 | 0.845 | 0.828 | 0.806 | 0.903 | 0.810 | 0.839 | 0.821 |
|  | *F_IS_* | 0.000 | 0.039 | 0.000 | 0.149 | 0.000 | 0.799 | 0.012 | 0.053 | 0.000 | 0.197 | 0.108 | 0.142 | 0.007 | 0.116 |
| Bissau S-form (3S) | *R_s_* | 5 | 14 | 11 | 9 | 9 | 8 | 8 | 9 | 7 | 6 | 11 | 12 | 6 | 9 |
|  | *H_e_* | 0.596 | 0.936 | 0.870 | 0.870 | 0.844 | **0.763*** | 0.774 | 0.860* | 0.798 | 0.562 | 0.907 | 0.854 | **0.795** | 0.802 |
|  | *F_IS_* | 0.000 | 0.198 | 0.250 | 0.000 | 0.013 | 0.583 | 0.030 | 0.321 | 0.000 | 0.110 | 0.127 | 0.024 | 0.267 | 0.148 |
| Bobo-Dioulasso (4) | *R_s_* | 5 | 16 | 9 | 9 | 9 | 7 | 7 | 8 | 7 | 9 | 12 | 7 | 6 | 9 |
|  | *H_e_* | 0.672 | 0.932 | 0.842 | 0.831 | 0.851 | **0.665*** | 0.569 | **0.846*** | 0.789 | **0.858*** | 0.902 | 0.793 | 0.742 | 0.792 |
|  | *F_IS_* | 0.000 | 0.005 | 0.178 | 0.098 | 0.133 | 0.466 | 0.000 | 0.337 | 0.216 | 0.295 | 0.026 | 0.159 | 0.000 | 0.147 |
| Goundry (5) | *R_s_* | 6 | 16 | 8 | 9 | 11 | 7 | 8 | 9 | 8 | 9 | 13 | 8 | 5 | 9 |
|  | *H_e_* | 0.620 | 0.946 | 0.825 | 0.869 | 0.860 | 0.696 | 0.593 | **0.813** | 0.825 | 0.816* | 0.907 | 0.836 | 0.736 | 0.796 |
|  | *F_IS_* | 0.137 | 0.017 | 0.000 | 0.102 | 0.031 | 0.089 | 0.020 | 0.199 | 0.000 | 0.313 | 0.028 | 0.066 | 0.155 | 0.089 |
| Okyereko (6) | *R_s_* | 8 | 13 | 10 | 9 | 10 | 9 | 6 | 9 | 5 | 10 | 12 | 10 | 5 | 9 |
|  | *H_e_* | 0.709 | 0.930 | 0.821 | 0.875 | 0.884 | **0.814*** | 0.576 | 0.787 | 0.796 | **0.852*** | 0.899 | 0.868 | 0.668 | 0.806 |
|  | *F_IS_* | 0.002 | 0.108 | 0.043 | 0.108 | 0.095 | 0.286 | 0.132 | 0.172 | 0.058 | 0.557 | 0.085 | 0.000 | 0.081 | 0.133 |
| Accra (7) | *R_s_* | 7 | 13 | 9 | 10 | 7 | 5 | 7 | 8 | 7 | 9 | 10 | 8 | 6 | 8 |
|  | *H_e_* | 0.653 | 0.909 | 0.875 | 0.857 | 0.794 | 0.285 | 0.717 | **0.848*** | 0.807 | **0.855*** | **0.849*** | 0.842 | 0.758 | 0.773 |
|  | *F_IS_* | 0.000 | 0.050 | 0.111 | 0.000 | 0.076 | 0.000 | 0.040 | 0.240 | 0.008 | 0.309 | 0.241 | 0.077 | 0.003 | 0.089 |
| Dassa (8) | *R_s_* | 5 | 12 | 8 | 9 | 9 | 8 | 8 | 8 | 5 | 9 | 11 | 8 | 5 | 8 |
|  | *H_e_* | 0.606 | 0.905 | 0.814 | **0.875*** | 0.841 | **0.756*** | 0.684 | 0.820 | 0.803 | **0.868*** | 0.863 | 0.784 | 0.639 | 0.789 |
|  | *F_IS_* | 0.108 | 0.099 | 0.169 | 0.289 | 0.085 | 0.603 | 0.000 | 0.209 | 0.138 | 0.441 | 0.066 | 0.182 | 0.323 | 0.209 |
| Kobape (9) | *R_s_* | 6 | 14 | 9 | 9 | 10 | 10 | 6 | 9 | 5 | 9 | 12 | 9 | 6 | 9 |
|  | *H_e_* | 0.644 | **0.916*** | 0.849 | 0.858 | 0.866 | **0.866*** | 0.525 | 0.802* | 0.774 | **0.873*** | 0.893 | 0.841 | 0.781 | 0.807 |
|  | *F_IS_* | 0.000 | 0.201 | 0.081 | 0.000 | 0.092 | 0.319 | 0.048 | 0.287 | 0.016 | 0.501 | 0.000 | 0.000 | 0.000 | 0.119 |
| Tiko (10) | R_s_ | 4 | 15 | 7 | 7 | 6 | 9 | 5 | 7 | 7 | 11 | 13 | 6 | 4 | 8 |
|  | H_e_ | 0.605 | 0.939 | 0.759 | 0.831 | 0.767 | **0.868*** | 0.561 | 0.821* | 0.802 | 0.859 | 0.919 | 0.677 | 0.615 | 0.771 |
|  | F_IS_ | 0.000 | 0.155 | 0.000 | 0.129 | 0.145 | 0.801 | 0.016 | 0.478 | 0.000 | 0.085 | 0.106 | 0.000 | 0.159 | 0.160 |
| Simbock (11) | *R_s_* | 4 | 13 | 8 | 8 | 6 | 8 | 5 | 8 | 7 | 9 | 10 | 7 | 5 | 7 |
|  | *H_e_* | 0.723 | 0.917 | 0.811 | 0.860 | 0.755 | **0.829*** | 0.568 | **0.808*** | 0.777 | 0.846* | **0.882*** | 0.776 | **0.742*** | 0.792 |
|  | *F_IS_* | 0.018 | 0.097 | 0.041 | 0.030 | 0.000 | 0.547 | 0.048 | 0.532 | 0.000 | 0.180 | 0.347 | 0.199 | 0.286 | 0.179 |
| Bayanga (12) | *R_s_* | 5 | 14 | 10 | 9 | 10 | 10 | 6 | 10 | 7 | 10 | 13 | 8 | 6 | 9 |
|  | *H_e_* | 0.611 | 0.924* | 0.873 | 0.839 | 0.876 | **0.899*** | 0.554 | **0.868*** | 0.794 | **0.797*** | **0.918** | 0.842 | 0.722 | 0.809 |
|  | *F_IS_* | 0.055 | 0.159 | 0.059 | 0.020 | 0.091 | 0.579 | 0.000 | 0.267 | 0.021 | 0.275 | 0.129 | 0.077 | 0.125 | 0.143 |
| Ngonamanga (13) | *R_s_* | 5 | 15 | 7 | 7 | 5 | 8 | 5 | 6 | 7 | 9 | 12 | 6 | 4 | 7 |
|  | *H_e_* | 0.679 | 0.921 | 0.795 | 0.738 | 0.614* | **0.822*** | 0.611 | **0.796*** | 0.763 | 0.871 | **0.896*** | 0.718 | 0.694 | 0.763 |
|  | *F_IS_* | 0.029 | 0.035 | 0.000 | 0.097 | 0.276 | 0.325 | 0.000 | 0.414 | 0.000 | 0.145 | 0.188 | 0.102 | 0.000 | 0.124 |

Table S3 (continued)

| Site |  | H242 | H128 | H249 | H119 | H555 | H127 | H577 | H750 | H59 | H88 | H758 | H93 | 45C | Mean |
| --- | --- | --- | --- | --- | --- | --- | --- | --- | --- | --- | --- | --- | --- | --- | --- |
| Bata (14) | *R_s_* | 4 | 14 | 8 | 5 | 5 | 7 | 4 | 8 | 7 | 9 | 11 | 7 | 5 | 7 |
|  | *H_e_* | 0.638 | 0.930 | 0.835 | 0.735 | 0.716 | **0.797*** | 0.547 | **0.849*** | 0.705 | 0.838* | 0.885 | 0.743 | 0.722 | 0.765 |
|  | *F_IS_* | 0.020 | 0.094 | 0.042 | 0.070 | 0.079 | 0.721 | 0.000 | 0.504 | 0.229 | 0.325 | 0.097 | 0.000 | 0.000 | 0.168 |
| Libreville (15) | *R_s_* | 5 | 7 | 7 | 9 | 6 | 4 | 4 | 6 | 6 | 6 | 6 | 11 | 5 | 6 |
|  | *H_e_* | 0.646 | 0.666 | 0.792 | 0.826 | 0.785 | 0.623 | 0.509 | **0.816*** | 0.746 | 0.731* | 0.669 | 0.862 | 0.593 | 0.713 |
|  | *F_IS_* | 0.000 | 0.000 | 0.000 | 0.059 | 0.000 | 0.109 | 0.000 | 0.455 | 0.047 | 0.361 | 0.000 | 0.000 | 0.026 | 0.081 |
| Benguia (16) | *R_s_* | 6 | 12 | 9 | 10 | 6 | 5 | 6 | 9 | 8 | 6 | 8 | 11 | 7 | 8 |
|  | *H_e_* | 0.669 | 0.894 | 0.853 | 0.876 | 0.771 | 0.438 | 0.580 | **0.845*** | 0.833 | 0.782 | 0.803 | 0.857 | 0.733 | 0.764 |
|  | *F_IS_* | 0.071 | 0.000 | 0.036 | 0.000 | 0.000 | 0.118 | 0.003 | 0.395 | 0.120 | 0.099 | 0.000 | 0.067 | 0.049 | 0.074 |
| Bakoumba (17) | *R_s_* | 6 | 12 | 9 | 9 | 6 | 5 | 7 | 7 | 8 | 6 | 8 | 9 | 5 | 8 |
|  | *H_e_* | 0.609 | 0.901 | 0.837 | 0.791 | 0.770 | 0.450 | 0.753 | **0.802*** | 0.818 | **0.762*** | 0.808 | 0.850 | 0.696 | 0.757 |
|  | *F_IS_* | 0.000 | 0.000 | 0.071 | 0.017 | 0.085 | 0.292 | 0.000 | 0.490 | 0.090 | 0.271 | 0.147 | 0.085 | 0.000 | 0.119 |
| Dienga (18) | *R_s_* | 5 | 11 | 7 | 8 | 7 | 6 | 5 | 7 | 6 | 7 | 10 | 10 | 5 | 7 |
|  | *H_e_* | 0.515 | 0.872 | 0.819 | 0.798 | 0.826 | 0.510 | 0.687 | **0.797*** | 0.781 | 0.709* | 0.836 | 0.824 | 0.722 | 0.746 |
|  | *F_IS_* | 0.000 | 0.134 | 0.186 | 0.000 | 0.000 | 0.129 | 0.029 | 0.414 | 0.032 | 0.217 | 0.043 | 0.000 | 0.077 | 0.097 |
| Cabinda (19) | *R_s_* | 6 | 12 | 10 | 9 | 6 | 4 | 6 | 8 | 7 | 6 | 10 | 10 | 6 | 8 |
|  | *H_e_* | 0.596 | 0.886 | 0.874 | 0.849 | 0.783 | **0.420*** | 0.736 | **0.817*** | 0.813 | 0.770* | 0.872 | 0.870* | 0.787 | 0.775 |
|  | *F_IS_* | 0.000 | 0.064 | 0.075 | 0.073 | 0.000 | 0.856 | 0.000 | 0.297 | 0.000 | 0.292 | 0.171 | 0.242 | 0.108 | 0.167 |
| Kikudo (20) | *R_s_* | 7 | 11 | 9 | 9 | 5 | 5 | 6 | 9 | 6 | 5 | 8 | 9 | 5 | 7 |
|  | *H_e_* | 0.726 | 0.906* | 0.838 | 0.848 | 0.699 | 0.418 | 0.647 | 0.848* | 0.849 | **0.762*** | 0.783 | 0.846 | 0.765 | 0.764 |
|  | *F_IS_* | 0.000 | 0.212 | 0.000 | 0.065 | 0.142 | 0.331 | 0.093 | 0.284 | 0.176 | 0.638 | 0.119 | 0.000 | 0.000 | 0.158 |
| Luanda (21) | *R_s_* | 5 | 11 | 8 | 5 | 4 | 8 | 4 | 8 | 6 | 6 | 8 | 6 | 4 | 6 |
|  | *H_e_* | 0.767 | 0.888* | 0.863 | 0.627 | 0.646 | **0.858*** | 0.492 | 0.801 | 0.681 | 0.697* | 0.850 | 0.762 | 0.698 | 0.741 |
|  | *F_IS_* | 0.000 | 0.181 | 0.000 | 0.000 | 0.191 | 0.295 | 0.168 | 0.303 | 0.000 | 0.266 | 0.000 | 0.045 | 0.000 | 0.111 |
| Cavaco (22) | *R_s_* | 4 | 12 | 7 | 6 | 6 | 7 | 2 | 7 | 7 | 6 | 10 | 6 | 4 | 7 |
|  | *H_e_* | 0.702 | 0.917* | 0.778 | 0.678 | 0.815 | **0.830*** | 0.157 | **0.836*** | 0.837 | 0.614 | 0.874 | 0.747 | 0.727 | 0.732 |
|  | *F_IS_* | 0.075 | 0.212 | 0.093 | 0.000 | 0.104 | 0.552 | 0.000 | 0.746 | 0.037 | 0.000 | 0.019 | 0.120 | 0.070 | 0.156 |
| Namibe (23) | *R_s_* | 6 | 12 | 6 | 4 | 6 | 6 | 2 | 5 | 7 | 5 | 8 | 5 | 4 | 6 |
|  | *H_e_* | 0.641 | 0.909 | 0.799 | 0.620 | 0.739 | **0.750*** | 0.091 | **0.736*** | 0.804 | 0.601 | 0.804 | 0.741 | 0.734 | 0.690 |
|  | *F_IS_* | 0.146 | 0.057 | 0.000 | 0.000 | 0.000 | 0.566 | 0.000 | 0.400 | 0.074 | 0.149 | 0.000 | 0.152 | 0.113 | 0.127 |
| Furvela (24) | *R_s_* | 3 | 6 | 5 | 6 | 5 | 5 | 4 | 7 | 8 | 5 | 6 | 9 | 5 | 6 |
|  | *H_e_* | **0.547*** | 0.624 | 0.417 | 0.685 | 0.742 | 0.393 | 0.394 | 0.718* | 0.814 | **0.604** | 0.757 | 0.794 | 0.666 | 0.627 |
|  | *F_IS_* | 0.432 | 0.109 | 0.006 | 0.171 | 0.161 | 0.152 | 0.000 | 0.272 | 0.071 | 0.251 | 0.148 | 0.084 | 0.231 | 0.161 |
| Mean | *R_s_* | 5 | 13 | 9 | 8 | 7 | 7 | 6 | 8 | 7 | 8 | 10 | 8 | 5 | 8 |
|  | *H_e_* | 0.640 | 0.894 | 0.819 | 0.809 | 0.792 | 0.692 | 0.575 | 0.822 | 0.793 | 0.778 | 0.859 | 0.807 | 0.731 | 0.770 |
|  | *F_IS_* | 0.044 | 0.090 | 0.068 | 0.061 | 0.074 | 0.433 | 0.026 | 0.332 | 0.058 | 0.268 | 0.101 | 0.076 | 0.093 | 0.133 |

Site: name of the locality surveyed and number according to Table 1 in parenthesis. *R_s_*: allele richness (El Mousadik & Petit 1996). *H_e_*: expected heterozygosity (Nei’s 1987 unbiased estimator). *F_IS_*: inbreeding coefficient (Weir & Cockerham 1984). In bold: significant HWE test. Asterisks indicate loci for which the presence of null alleles was detected by Micro-Checker.

Table S4. Power and accuracy of the Bayesian clustering analysis implemented by STRUCTURE (Pritchard et al. 2000) to detect M and S form simulated individuals (*N*=100 for each form)

| *T_q_* | Cluster | *N*_cor_ | *N_ass_* | Power | Accuracy |
| --- | --- | --- | --- | --- | --- |
| 0.90 | M-form | 59 | 63 | 0.59 | 0.94 |
|  | S-form | 58 | 62 | 0.58 | 0.94 |
| 0.80 | M-form | 95 | 121 | 0.95 | 0.79 |
|  | S-form | 94 | 125 | 0.94 | 0.75 |
| 0.75 | M-form | 100 | 142 | 1.00 | 0.70 |
|  | S-form | 99 | 150 | 0.99 | 0.66 |
| 0.70 | M-form | 100 | 167 | 1.00 | 0.60 |
|  | S-form | 100 | 180 | 1.00 | 0.56 |

Legend: *T_q_*: probability threshold for assignment to each cluster (M-form or S-form); *N_cor_*: number of correctly assigned individuals; *N_ass_*: total number of assigned individuals. Power: proportion of individuals in a group that were correctly identified. Accuracy: proportion of an identified group that truly belongs to that group.

Table S5: Isolation by distance model regressions of *F_ST_*/(1-*F_ST_*) on logarithm of distance

|  | *N* | Distance range (km) | Intercept | Slope | *P* | *r*^2^ |
| --- | --- | --- | --- | --- | --- | --- |
| All samples | 25 | 1^a^ - 7,197 | 0.0047 | 0.0007 | <0.001 | 0.0932 |
| M-form | 16 | 18 - 5,317 | -0.0206 | 0.0066 | <0.001 | 0.3174 |
| S-form | 9 | 36 - 7,141 | -0.0211 | 0.0072 | 0.014 | 0.4290 |
| S-form West^b^ | 8 | 30 - 4,250 | -0.0033 | 0.0041 | 0.046 | 0.2844 |

*N*: number of samples included in each regression analysis; *P*: p-value of Mantel tests; *r*^2^: coefficient of determination. ^a^ The two sympatric M- and S-forms from Antula, Guinea Bissau were considered as 1km apart to avoid zero-values in the logarithmic transformation. ^b^ The east African sample (Furvela, Mozambique) was removed from this regression.

Table S6. Pair-wise estimates of *F_ST_* (below diagonal) and geographic distance (above diagonal, in kilometres) between sampling sites

|  | [1] | [2] | [3M] | [3S] | [4] | [5] | [6] | [7] | [8] | [9] | [10] | [11] | [12] | [13] | [14] | [15] | [16] | [17] | [18] | [19] | [20] | [21] | [22] | [23] | [24] |
| --- | --- | --- | --- | --- | --- | --- | --- | --- | --- | --- | --- | --- | --- | --- | --- | --- | --- | --- | --- | --- | --- | --- | --- | --- | --- |
| [1] |  | 18 | 199 | 199 | 1187 | 1487 | 1812 | 1839 | 1978 | 2131 | 2886 | 3096 | 3627 | 3188 | 3216 | 3402 | 3692 | 3691 | 3678 | 4064 | 4134 | 4476 | 4881 | 5210 | 7197 |
| [2] | 0.013 |  | 203 | 203 | 1166 | 1462 | 1795 | 1822 | 1963 | 2116 | 2868 | 3079 | 3611 | 3114 | 3141 | 3346 | 3678 | 3669 | 3656 | 4041 | 4112 | 4473 | 4894 | 5217 | 7061 |
| [3M] | 0.005 | 0.009 |  | 0 | 1237 | 1557 | 1808 | 1839 | 2010 | 2163 | 2966 | 3153 | 3658 | 3325 | 3280 | 3524 | 3779 | 3783 | 3774 | 4171 | 4249 | 4569 | 5000 | 5317 | 7140 |
| [3S] | 0.014 | 0.016 | 0.002 |  | 1238 | 1558 | 1809 | 1840 | 2011 | 2164 | 2967 | 3154 | 3659 | 3326 | 3281 | 3525 | 3780 | 3784 | 3775 | 4172 | 4250 | 4570 | 5001 | 5318 | 7141 |
| [4] | 0.009 | 0.023 | **0.012** | **0.019** |  | 355 | 759 | 770 | 808 | 959 | 1720 | 1922 | 2448 | 2075 | 2100 | 2273 | 2573 | 2539 | 2528 | 2922 | 2999 | 3309 | 3750 | 4067 | 5910 |
| [5] | 0.012 | 0.023 | **0.010** | 0.012 | 0.001 |  | 789 | 787 | 651 | 782 | 1509 | 1707 | 2210 | 1798 | 1826 | 2015 | 2302 | 2299 | 2286 | 2672 | 2749 | 3064 | 3503 | 3819 | 5705 |
| [6] | **0.011** | **0.030** | **0.016** | **0.027** | **0.017** | **0.014** |  | 43 | 405 | 500 | 1230 | 1435 | 1968 | 1560 | 1585 | 1804 | 2052 | 2053 | 2043 | 2442 | 2516 | 2823 | 3263 | 3577 | 5383 |
| [7] | **0.034** | **0.042** | **0.030** | **0.034** | **0.020** | **0.022** | **0.025** |  | 365 | 457 | 1191 | 1390 | 1924 | 1476 | 1502 | 1687 | 1986 | 1983 | 1970 | 2366 | 2450 | 2750 | 3187 | 3534 | 5377 |
| [8] | **0.017** | 0.021 | **0.014** | **0.021** | 0.010 | 0.005 | 0.015 | **0.027** |  | 153 | 907 | 1117 | 1649 | 1244 | 1273 | 1456 | 1736 | 1735 | 1724 | 2114 | 2190 | 2540 | 2976 | 3285 | 5115 |
| [9] | 0.012 | 0.025 | **0.017** | **0.029** | **0.014** | **0.011** | **0.010** | **0.032** | 0.015 |  | 754 | 964 | 1496 | 1072 | 1101 | 1281 | 1577 | 1575 | 1563 | 1949 | 2019 | 2337 | 2770 | 3085 | 4953 |
| [10] | **0.038** | **0.031** | **0.040** | **0.045** | **0.039** | **0.037** | **0.045** | **0.069** | **0.037** | **0.040** |  | 238 | 779 | 269 | 298 | 472 | 800 | 794 | 780 | 1155 | 1226 | 1555 | 1972 | 2305 | 4190 |
| [11] | **0.032** | **0.042** | **0.041** | **0.048** | **0.049** | **0.045** | **0.046** | **0.079** | **0.048** | **0.042** | 0.009 |  | 541 | 263 | 285 | 441 | 643 | 647 | 641 | 1046 | 1116 | 1425 | 1855 | 2170 | 3996 |
| [12] | 0.004 | **0.016** | 0.007 | **0.015** | 0.013 | 0.010 | **0.009** | **0.038** | 0.015 | **0.013** | **0.021** | **0.026** |  | 724 | 728 | 807 | 592 | 637 | 660 | 1044 | 1095 | 1344 | 1751 | 2070 | 3597 |
| [13] | **0.041** | **0.059** | **0.047** | **0.056** | **0.050** | **0.047** | **0.052** | **0.062** | **0.052** | **0.052** | 0.032 | **0.023** | 0.036 |  | 30 | 204 | 582 | 567 | 546 | 902 | 973 | 1293 | 1729 | 2047 | 3986 |
| [14] | **0.037**  **(0.041)** | **0.047**  **(0.052)** | **0.040**  **(0.045)** | **0.051**  **(0.055)** | **0.051**  **(0.055)** | **0.046**  **(0.050)** | **0.048**  **(0.054)** | **0.076**  **(0.082** | **0.049**  **(0.053)** | **0.040**  **(0.044)** | 0.020  (0.021) | **0.009**  (0.009) | **0.021**  **(0.024)** | **0.011**  (0.011) |  | 185 | 561 | 544 | 523 | 873 | 943 | 1266 | 1702 | 2018 | 3965 |
| [15] | **0.070** | **0.096** | **0.068** | **0.081** | **0.073** | **0.074** | **0.049** | **0.062** | **0.076** | **0.066** | **0.113** | **0.110** | **0.067** | **0.110** | **0.117 (0.125)** |  | 496 | 469 | 441 | 760 | 833 | 1149 | 1577 | 1895 | 3883 |
| [16] | **0.032** | **0.055** | **0.032** | **0.038** | **0.027** | **0.032** | **0.027** | **0.015** | **0.037** | **0.036** | **0.070** | **0.078** | **0.036** | **0.069** | **0.082 (0.089)** | **0.043** |  | 52 | 87 | 460 | 519 | 804 | 1236 | 1555 | 3404 |
| [17] | **0.041** | **0.060** | **0.035** | **0.042** | **0.034** | **0.039** | **0.034** | **0.018** | **0.041** | **0.046** | **0.080** | **0.089** | **0.044** | **0.075** | **0.087 (0.093)** | **0.050** | 0.005 |  | 36 | 428 | 488 | 784 | 1208 | 1529 | 3421 |
| [18] | **0.041** | **0.064** | **0.035** | **0.044** | **0.038** | **0.042** | **0.036** | **0.028** | **0.042** | **0.046** | **0.083** | **0.089** | **0.046** | **0.080** | **0.089 (0.095)** | **0.045** | **0.013** | **0.007** |  | 419 | 482 | 811 | 1244 | 1557 | 3448 |
| [19] | **0.038** | **0.052** | **0.033** | **0.038** | **0.029** | **0.032** | **0.031** | **0.020** | **0.034** | **0.040** | **0.069** | **0.069** | **0.042** | **0.068** | **0.075 (0.082)** | **0.061** | **0.017** | **0.015** | **0.019** |  | 79 | 397 | 832 | 1148 | 3232 |
| [20] | **0.038** | **0.055** | **0.036** | **0.041** | **0.033** | **0.037** | **0.044** | **0.024** | **0.043** | **0.049** | **0.085** | **0.084** | **0.048** | **0.072** | **0.091 (0.097)** | **0.060** | **0.014** | **0.017** | **0.032** | **0.027** |  | 335 | 761 | 1078 | 3130 |
| [21] | **0.059** | **0.070** | **0.060** | **0.067** | **0.076** | **0.067** | **0.061** | **0.086** | **0.073** | **0.063** | **0.055** | **0.045** | **0.045** | **0.046** | **0.038 (0.041)** | **0.124** | **0.084** | **0.101** | **0.111** | **0.091** | **0.096** |  | 441 | 762 | 2865 |
| [22] | **0.044** | **0.058** | **0.049** | **0.057** | **0.060** | **0.056** | **0.049** | **0.089** | **0.066** | **0.054** | **0.048** | **0.040** | **0.030** | **0.045** | **0.024 (0.026)** | **0.103** | **0.077** | **0.094** | **0.093** | **0.085** | **0.089** | **0.024** |  | 321 | 2619 |
| [23] | **0.070** | **0.079** | **0.071** | **0.081** | **0.087** | **0.075** | **0.074** | **0.107** | **0.089** | **0.072** | **0.070** | **0.061** | **0.056** | **0.068** | **0.047 (0.050)** | **0.136** | **0.100** | **0.115** | **0.117** | **0.103** | **0.109** | **0.037** | **0.019** |  | 2606 |
| [24] | **0.098** | **0.132** | **0.098** | **0.107** | **0.095** | **0.102** | **0.094** | **0.090** | **0.116** | **0.109** | **0.135** | **0.145** | **0.101** | **0.123** | **0.143 (0.150)** | **0.110** | **0.064** | **0.077** | **0.072** | **0.084** | **0.100** | **0.141** | **0.111** | **0.146** |  |

Sample numbers are according to Table S1 and Figure 1. In sample [14] (Bata) the values in parenthesis are the estimates obtained after removing two misidentified individuals (one *An. melas* and one putative hybrid between *An. gambiae s.s.* M-form and *An. melas,* see Results). Values in bold represent significant *F_ST_* values after correction of the nominal significance value by the sequential Bonferroni procedure (see Methods).

Figure S1. Plots between genetic differentiation and expected heterozygosity to detect candidate microsatellite loci under selection according to the method implemented in LOSITANT (Antao et al. 2008). A:infinite alleles model; B: stepwise mutation model

A


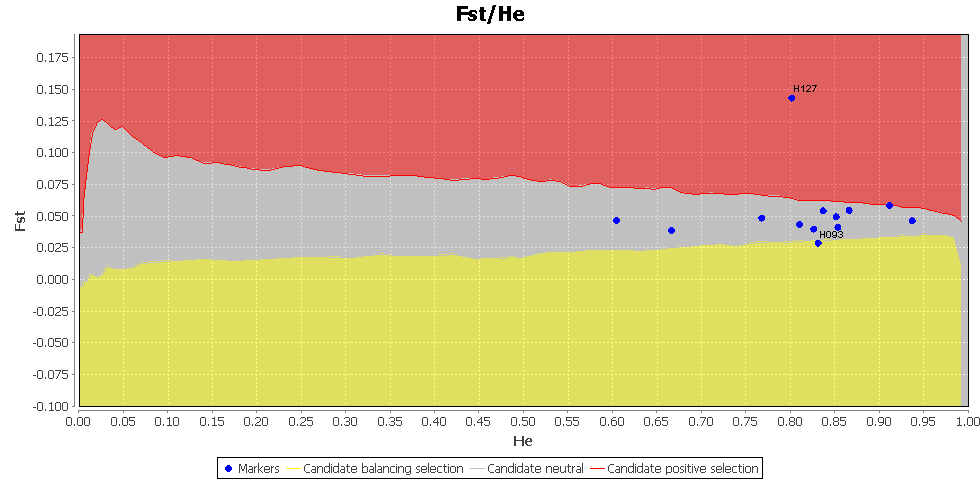


B


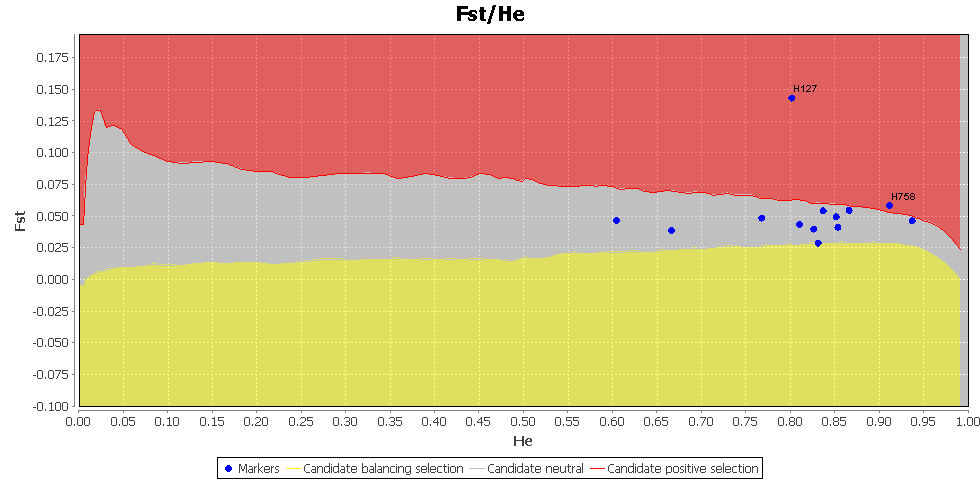


Figure S2. Graphics of Evanno’s *ΔK* for the different Bayesian clustering analyses implemented by structure

**All samples (Figure 1, A and B)**

**M-form samples (Figure 1, M-form)**

**S-form samples (Figure 1, S-form)**

Figure S3. Bayesian clustering analysis implemented by STRUCTURE (Pritchard et al. 2000) and spatially explicit analyses implemented by TESS (Chen et al. 2007), performed with 10 microsatellite loci.


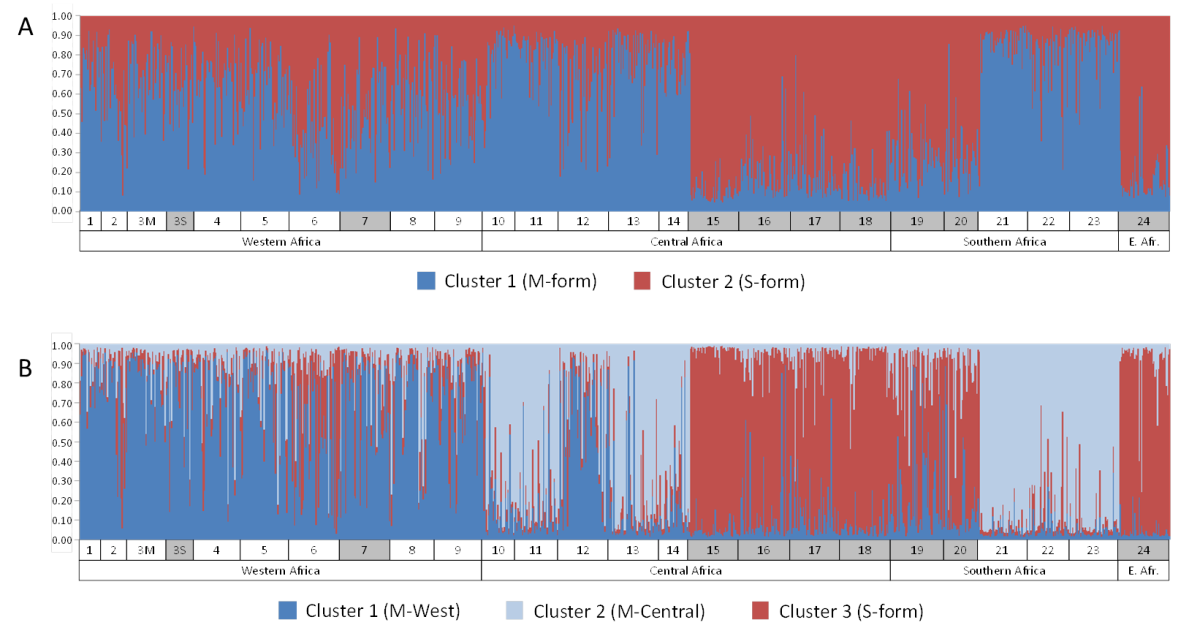


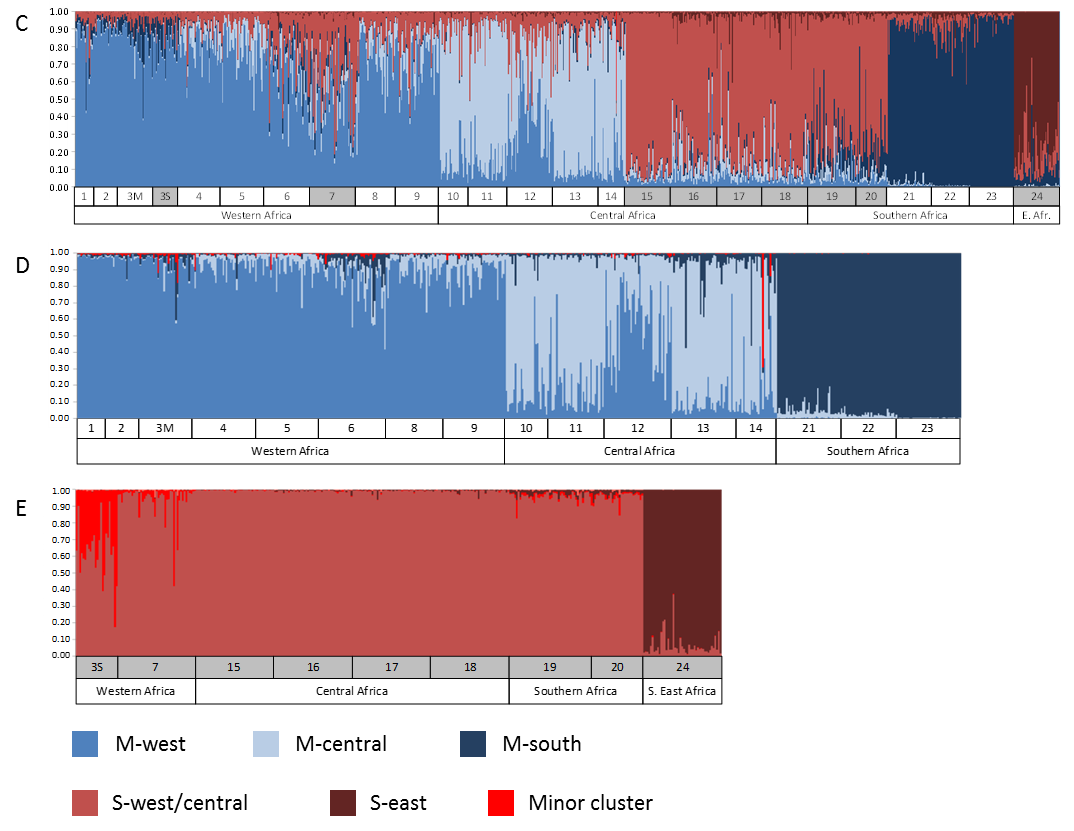


Legend: These analyses were conducted excluding loci AG3H88, AG3H127 and AG3H750, which displayed the most heterozygote deficits with evidence for the presence of null alleles and/or selection. Localities sampled are numbered according to Table S1 in a northwest–southeast direction along the X-axis (Figure 1). White boxes indicate M-form and grey boxes indicate S-form samples as determined by the IGS^581^ marker. Y-axis: probability of assignment to each cluster. Each column corresponds to the multilocus genotype of a single individual partitioned into colours representing the probability of assignment to each cluster. A: STRUCTURE analysis with all samples, *K*=2; B: STRUCTURE analysis with all samples, *K*=3; C: TESS analysis with all samples; D: TESS analysis for M-form samples (*K_max_* = 4); E: TESS analysis for S-form samples (*K_max_* = 4). All TESS analyses were performed under the CAR model (see Methods)

Figure S4. Plots of DIC values (Y-axis) against *K_max_* (X-axis) obtained for TESS analysis (Chen *et al.* 2007) under two admixture models (CAR and BYM)

**All samples**


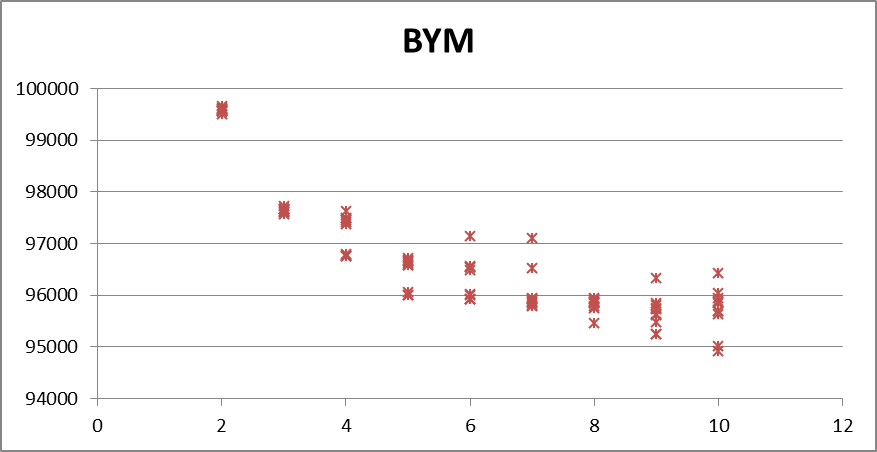
**
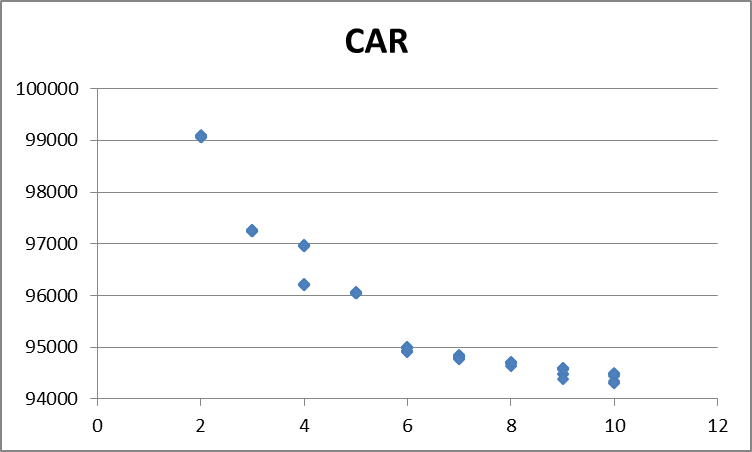
**

**M-form**


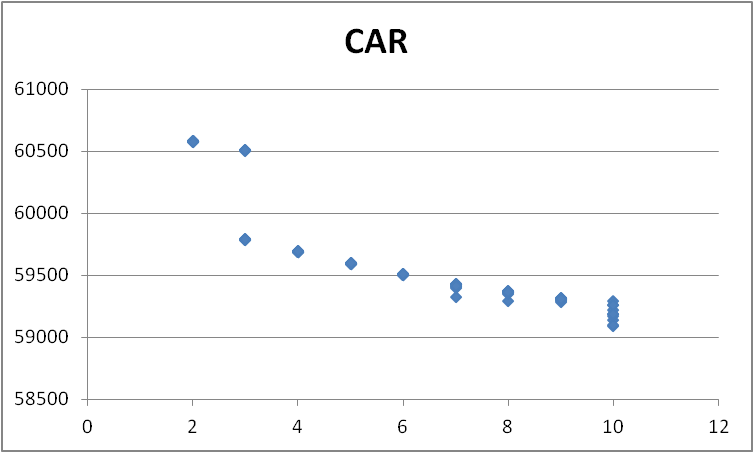

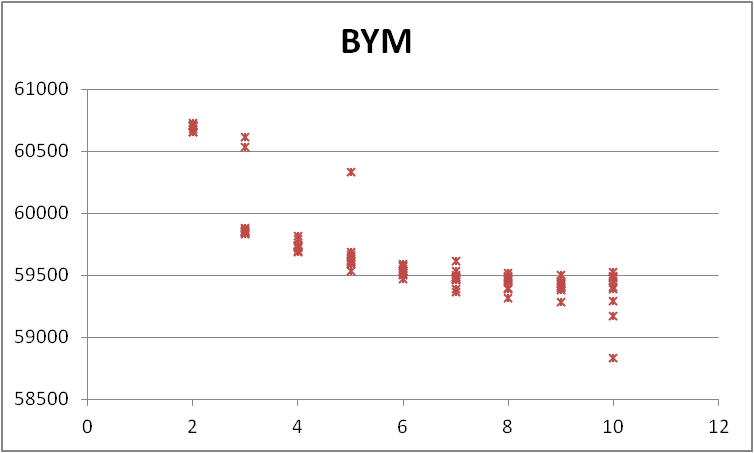


**S-form**

**
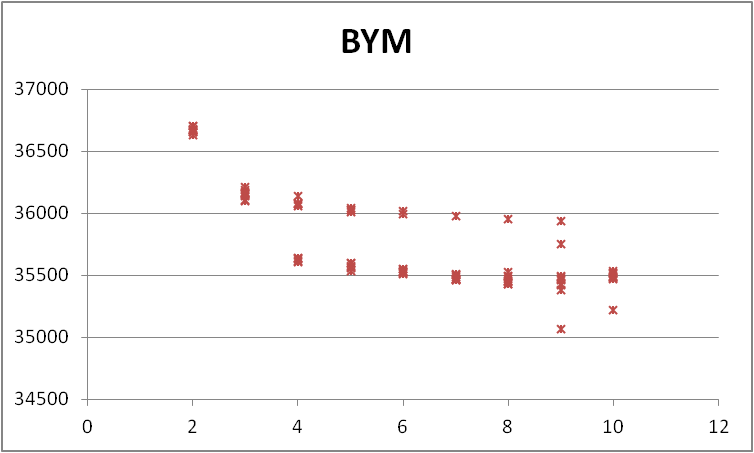

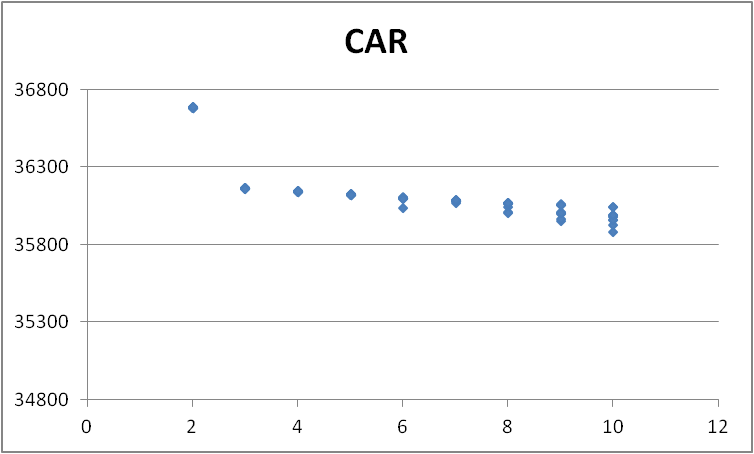
**
